# Direct Immobilization of Living Poly(2-ethyl-2-oxazoline) Chains onto Mesoporous Silica: A Simplified Grafting-To Strategy for Hybrid Organic–Inorganic Materials

**DOI:** 10.3390/ma19173775

**Published:** 2026-09-04

**Authors:** Marcelina Bochenek, Margarita Popova, Natalia Oleszko-Torbus, Agnieszka Kowalczuk, Alicja Utrata-Wesołek, Violeta Mitova, Neli Koseva, Elżbieta Grządka, Jolanta Orzeł, Barbara Mendrek

**Affiliations:** 1 Centre of Polymer and Carbon Materials, Polish Academy of Sciences, M. Curie-Sklodowskiej 34, 41-819 Zabrze, Poland; mbochenek@cmpw-pan.pl (M.B.); noleszko@cmpw-pan.pl (N.O.-T.); akowalczuk@cmpw-pan.pl (A.K.); autrata@cmpw-pan.pl (A.U.-W.); 2 Institute of Organic Chemistry with Centre of Phytochemistry, Bulgarian Academy of Sciences, 1113 Sofia, Bulgaria; margarita.popova@orgchm.bas.bg; 3 Institute of Polymers, Bulgarian Academy of Sciences, 1113 Sofia, Bulgaria; mitova@polymer.bas.bg (V.M.); koseva@polymer.bas.bg (N.K.); 4 Physical Chemistry Department, Faculty of Chemistry, Institute of Chemical Sciences, Maria Curie-Skłodowska University, M. Curie-Sklodowska Sq. 3, 20-031 Lublin, Poland; elzbieta.grzadka@mail.umcs.pl (E.G.); jolanta.orzel@umcs.pl (J.O.)

**Keywords:** mesoporous silica particles, poly(2-ethyl-2-oxazoline), grafting-to, hybrid material

## Abstract

The development of straightforward and efficient strategies for the preparation of polymer-functionalized mesoporous silica remains an important challenge in the design of advanced hybrid materials. Herein, we report a novel and simplified approach to the covalent functionalization of mesoporous silica particles (MSP) with poly(2-ethyl-2-oxazoline) (PEtOx), based on the direct termination of living cationic polymer chains by amino groups immobilized on the silica surface. In contrast to conventional grafting-to methods, the proposed strategy eliminates the need for polymer end-group functionalization while avoiding the synthetic complexity associated with surface-initiated polymerization. Well-defined PEtOx chains with number-average molar masses of 5000 and 7500 g mol^−1^ were synthesized by cationic ring-opening polymerization (CROP) and subsequently grafted onto amino-functionalized MSP. Successful covalent immobilization of the polymer was confirmed by Fourier-transform infrared spectroscopy (FT-IR), elemental analysis, thermogravimetric analysis (TGA), scanning electron microscopy (SEM), transmission electron microscopy (TEM), contact angle measurements, and nitrogen adsorption–desorption studies. The modification preserved the ordered mesoporous architecture while increasing particle hydrophilicity and decreasing the specific surface area and pore volume due to polymer incorporation. Shorter polymer chains exhibited higher grafting efficiency than higher-molar-mass analog, indicating that steric hindrance is an important factor influencing the grafting process. The presented methodology provides a versatile and experimentally accessible platform for the preparation of well-defined poly(2-oxazoline)-functionalized mesoporous silica with tunable physicochemical properties. Owing to the combination of a porous inorganic framework and a polymer shell, the obtained hybrid materials represent promising candidates for drug delivery, adsorption technologies, and other advanced biomedical and environmental applications.

## 1. Introduction

Mesoporous silica particles (MSP), particularly at the nanoscale, have emerged as one of the most extensively investigated inorganic materials in modern medical and biological research, attracting considerable scientific interest due to their unique structural features and broad potential in drug delivery, imaging, catalysis, and theranostic applications [1,2,3,4]. Their intrinsic properties, including a large specific surface area, a well-developed porous structure enabling high loading capacity, and facile surface functionalization, make them highly versatile platforms for the development of advanced systems. The functionalization of mesoporous silica particles with polymers is currently being intensively explored and developed, as it enhances their colloidal stability by introducing steric hindrance and suppressing particle aggregation, which is particularly crucial for their applications in diagnostics and drug delivery [5,6,7,8,9,10,11,12]. Polymers can be tethered to silica particles through physical interactions or by covalent attachment. The latter approach provides significantly greater stability of the polymer coating on the silica particle surface. The methods for the preparation of mesoporous silica particles covalently bonded with polymers are analogous to those applied to planar, non-porous silica surfaces and comprise two main approaches: the grafting-to and grafting-from strategies, which correspond to coupling reactions and surface-initiated polymerization, respectively [13,14]. Although the grafting-from approach typically enables higher grafting densities and the formation of polymer brush architectures, the principal advantage of the grafting-to method lies in the straightforward preparation of hybrid particles and the possibility of characterizing the synthesized polymer before grafting. Most published studies focus on the preparation of MSP covalently bonded with thermo- [9,15] or pH-sensitive [16,17,18,19] polymers. A variety of polymer-grafted silica particles have been reported, including those based on poly(N-isopropylacrylamide) [9], poly(oligo(ethylene glycol) methacrylates) [15], poly(acrylic acid) [16], poly(dimethylaminoethyl methacrylate) [17], poly(2-vinylpyridine) [19], as well as poly(ethylene glycol) [20], which is regarded as the gold standard polymer in medical applications. Poly(2-oxazoline)s (POx), referred to as pseudopeptides [21,22,23], are widely acknowledged for their biocompatibility and low cytotoxicity, as confirmed in numerous cell lines over a wide concentration range [24,25,26]. Consequently, they are regarded as a promising alternative to poly(ethylene glycol), additionally offering thermoresponsivity in aqueous solutions [27,28,29,30,31], which may further broaden their applicability in biological systems. Various silica-based materials, including monodispersed silica nanoparticles, mesoporous silica, and silica-based nanorods, have been covalently functionalized with poly(2-methyl-2-oxazoline) (PMeOx) or poly(2-ethyl-2-oxazoline) (PEtOx) using either grafting-from [32,33,34] or grafting-to [7,8,35,36,37] approaches. The strategies reported for the preparation of POx covalently bonded with silica-based materials are associated with certain synthetic challenges. In the case of the grafting-from approach, carefully optimized conditions are required to successfully carry out cationic ring-opening polymerization (CROP) of 2-oxazolines directly from the surface of silica materials. In turn, the grafting-to strategy relies on the presence of complementary functional groups both within the POx chains and on the silica surface, which may require additional synthetic effort and careful functionalization procedures. These limitations highlight the need for the development of more straightforward and efficient approaches for the preparation of POx-functionalized silica materials, which constitutes the main motivation for the present study.

Here, to the best of our knowledge, we demonstrate for the first time a novel and simplified approach to preparing POx covalently bonded to mesoporous silica particles. The proposed strategy involves the CROP of 2-oxazoline, in which the living polymer chains are terminated directly by amino groups present on the surface of silica particles. In contrast to previously reported approaches, the presented method does not require additional polymer functionalization procedures or highly restrictive grafting conditions, which considerably simplifies the overall synthetic pathway and facilitates the preparation of hybrid materials. PEtOx was selected as a model polymer due to its relatively facile synthesis, literature-confirmed biocompatibility, and low toxicity, which may constitute an important advantage in the context of future applications. Mesoporous silica particles were selected as the inorganic support owing to their highly porous structure, large specific surface area, and broad application potential, particularly in CO_2_ adsorption and drug delivery systems. The successful attachment of PEtOx to the MSP surface was experimentally demonstrated, and the influence of polymer molar mass on the properties of the obtained mesoporous hybrid materials was investigated. The PEtOx-grafted silica particles were comprehensively characterized using a combination of analytical techniques, including FT-IR spectroscopy, nitrogen adsorption–desorption analysis, elemental analysis, SEM, and TEM. The developed simplified method for attachment of PEtOx chains onto MSP enabled the preparation of well-defined organic–inorganic hybrid material with tunable properties for future functional applications.

## 2. Materials and Methods

### 2.1. Materials

2-Ethyl-2-oxazoline (EtOx, >99%, Sigma-Aldrich, Saint Louis, MO, USA) was distilled under reduced pressure, dried over CaH_2_, and distilled again. Acetonitrile (ACN, >99%, Merck, Darmstadt, Germany) was dried over CaH_2_ and distilled under a dry argon atmosphere. Toluene (Sigma-Aldrich, Saint Louis, MO, USA) was dried via azeotropic distillation. Methyl 4-nitrobenzenesulfonate (99% Sigma-Aldrich, Saint Louis, MO, USA), p-xylene (99%, VWR, Radnor, PA, USA), methanol (gradient grade for HPLC, ChemSolute, Renningen, Germany), acetone (min 99.5%, ChemSolve, Łódź, Poland), tetraethylorthosilicate (TEOS, 98% Fisher Scientific, Loughborough, UK), Pluronic P123 (≥98%, Sigma-Aldrich Chemie, Schnelldorf, Germany), (3-aminopropyl)triethoxysilane (APTES ≥ 98%, Sigma-Aldrich, Saint Louis, MO, USA), and hydrochloric acid (37%, Valerus, Sofia, Bulgaria) were used as received.

### 2.2. Synthesis of Mesoporous Silica Particles (MSP)

The synthesis of MSP (SBA-15) was performed by hydrothermal synthesis according to a previously reported method [38]. Briefly, the procedure was as follows: Pluronic P123 (6 g, 1.0 mmol) was dissolved in 180 mL distilled water and 18.5 g HCl (37%) at room temperature under vigorous stirring until the template was completely dissolved. TEOS (12 g), used as the silica source, was then added to the solution, and the mixture was stirred at 35 °C for 24 h. The obtained gel was transferred to a Teflon-lined stainless-steel autoclave and heated at 95 °C for 24 h. The resulting suspension was filtered, washed with distilled water, and dried. Template removal was carried out by calcination in air at 290 °C for 2 h, followed by a final dwell at 550 °C for 5 h with a heating rate of 1 °C/min.

### 2.3. Synthesis of Amino-Functionalized Mesoporous Silica Particles (MSP-NH_2_)

Surface modification of MSP with amino groups was achieved by grafting APTES onto the silica surface [39]. In a typical procedure, 1.00 g of the silica support, previously dried at 150 °C for 3 h, was dispersed in 35 mL of anhydrous toluene, followed by the addition of 15 mL of APTES. The reaction mixture was stirred at 60 °C for 24 h. After completion of the reaction, the resulting solid was recovered by filtration and sequentially washed with toluene, methanol, and distilled water to remove unreacted silane and residual solvent. The amino-functionalized material was subsequently dried at room temperature under high vacuum. The resulting sample was denoted as MSP-NH_2_ and contained 1.12 mmol of amine groups per 1 g of silica. Prior to the grafting of PEtOx, the MSP-NH_2_ was dried again in the reactor under high vacuum at room temperature for 48 h.

### 2.4. Synthesis of Poly(2-ethyl-2-oxazoline) (PEtOx1 and PEtOx2)

CROP of EtOx was carried out in two parallel polymerizations to obtain homopolymers with different molar masses. The theoretical degree of polymerization (DP) was set at 50 for PEtOx1 and 75 for PEtOx2. Methyl 4-nitrobenzenesulfonate (2 × 10^−4^ mol, 0.043 g) dissolved in anhydrous acetonitrile was placed in a reactor under a dry argon atmosphere, and next, the monomer was added (the concentration of the monomer in acetonitrile in both polymerizations was kept constant and was equal 1.43 mol/L). The polymerization was carried out at 70 °C under a dry argon atmosphere until complete conversion of EtOx, as determined by gas chromatography (GC) using p-xylene as an internal standard.

### 2.5. Grafting of PEtOx to MSP (MSP-PEtOx1 and MSP-PEtOx2)

Upon completion of the EtOx polymerization, the resulting polymer solution was directly added under reduced pressure to the previously dried MSP-NH_2_ (0.01 g), without exposure to air and moisture. The grafting reaction proceeded for 4 days at room temperature (the reaction time was extended because the grafting process was carried out without stirring to avoid altering the silica structure). Subsequently, methanol was added and the mixture was centrifuged at 10,000 rcf for 10 min. The resulting precipitate was repeatedly (5 times) washed with methanol and centrifuged after each washing step to remove unreacted polymer chains, yielding the hybrid particles. In the control experiment, PEtOx terminated with n-propylamine (M_n_ = 5500 g mol^−1^) was mixed with MSP-NH_2_. Reaction and washing conditions equivalent to those used for the living PEtOx chains were applied.

### 2.6. Methods

#### 2.6.1. Gas Chromatography (GC)

The conversion of EtOx was determined by GC Shimadzu Nexis GC 2030 (Shimadzu Corporation, Kyoto, Japan), equipped with two chromatographic lines with FID and BID-2030 detectors and TR-5 (30 m × 0.32 mm ID; 0.25 μm, Thermo Scientific, Waltham, MA, USA) and SH-I-17 (30 m × 0.32 mm ID; 0.50 μm, Shimadzu Corporation, Kyoto, Japan) columns.

#### 2.6.2. Gel Permeation Chromatography with Multiangle Laser Light Scattering (GPC-MALLS)

The molar mass and molar mass dispersity (Đ) of PEtOx were determined using a gel permeation chromatography with multiangle laser light scattering detector (DAWN HELEOS, Wyatt Technologies, Santa Barbara, CA, USA, λ = 658 nm) and a refractive index detector (Dn-3010 RI WGE DR Bures, Dallgow, Germany, λ = 620 nm). Measurements were carried out in N,N′-dimethylformamide (DMF) (POCH, Gliwice, Poland) with 5 mmol L^−1^ of LiBr and a flow rate of 1 mL min^−1^. The following set of GRAM columns were used: 100 Å, 1000 Å and 3000 Å (Polymer Standards Service, Mainz, Germany).

#### 2.6.3. Infrared Spectroscopy

The FT-IR spectra of the samples were collected at room temperature using a Thermo Nicolet 8700 spectrometer equipped with a Smart Orbit™ diamond ATR accessory and a DTGS detector (Thermo Fisher Scientific, Madison, WI, USA). Measurements were performed in the spectral range of 4000–400 cm^−1^ with a resolution of 4 cm^−1^. Each spectrum was obtained by averaging 64 scans to improve the signal-to-noise ratio. Post-acquisition data processing included ATR correction, baseline adjustment, and intensity normalization.

#### 2.6.4. Elemental Analysis

Elemental analysis of the samples was performed using an EuroEA3000 CHNS Analyzer (EuroVector, Pavia, Italy). Each analysis was conducted in triplicate, and the mean values are reported.

#### 2.6.5. Thermogravimetric Analysis (TGA)

TGA measurements were performed with TGA/DSC1 (Mettler-Toledo AG, Greifensee, Switzerland) equipment. Samples were heated from 40 to 800 °C with heating rate 20 °C/min. Measurements were performed under N_2_ atmosphere with flow rate 50 mL/min.

#### 2.6.6. Nitrogen Adsorption–Desorption

The specific surface area and pore size of the investigated samples were determined from nitrogen adsorption–desorption isotherms measured at 77 K. Prior to analysis, the samples were degassed at 333 K to remove adsorbed moisture and gases. The low-temperature nitrogen adsorption–desorption measurements were carried out using a volumetric method with an ASAP 2420 analyzer (Micromeritics Corp., Norcross, GA, USA). The specific surface area (S_BET_), Langmuir surface area, average pore diameter (D), and total pore volume (V_p_) of the materials were calculated according to the Brunauer–Emmett–Teller (BET) method [40].

#### 2.6.7. Scanning Electron Microscopy-Energy-Dispersive X-Ray Spectroscopy (SEM-EDS)

The morphology and elemental composition of the samples were examined using a Quanta 3D FEG scanning electron microscope equipped with an EDS detector (FEI, Hillsboro, OR, USA). SEM micrographs were acquired at an accelerating voltage of 5.00 kV with a magnification of 100,000×. For EDS analysis, the accelerating voltage was set to 20.00 kV and micrographs were recorded at a magnification of 1000. Each analysis was performed four times, and the mean values and corresponding standard deviations are reported.

#### 2.6.8. Transmission Electron Microscopy (TEM)

Samples were dried, finely ground with an agate mortar, and dispersed in 99.8 *v*/*v* % ethanol (POCH, Gliwice, Poland) to form a slurry. After 10 s of ultrasonic homogenization (Omni Sonic Ruptor 400, Omni International, Kennesaw, GA, USA), the suspension was deposited onto 300-mesh copper grids coated with carbon-reinforced lacey formvar (Ted Pella, Redding, CA, USA) and air-dried. Grids were mounted on a single-tilt holder and examined using a Titan G2 60–300 kV high-resolution TEM (FEI, Hillsboro, OR, USA) at 300 kV.

#### 2.6.9. Contact Angle

A suspension containing 4 mg of bare or modified silica particles in acetone was prepared. The suspension was thoroughly shaken, and 30 μL of the suspension was drop-casted onto a previously degreased silicon wafer (Cemat Silicon S.A., Bydgoszcz, Poland; with a 3 nm SiO_2_ layer). The samples were then air-dried for 2 days. The wettability of the prepared samples was measured using a CAM101 goniometer (KSV Instruments, Helsinki, Finland). The contact angle measurements were performed using the sessile drop method. A water droplet was placed on the surface and was registered for 30 s. The measurements were performed at room temperature. The contact angle values were reported as the average of three measurements.

## 3. Results and Discussion

### 3.1. Grafting of the Polymers to MSP

The main objective of this study was to develop a straightforward strategy for the covalent immobilization of PEtOx onto MSP by exploiting the intrinsic reactivity of living polymer chains generated during CROP. In contrast to previously reported grafting-to approaches, which generally require the prior synthesis of end-functionalized polymers followed by coupling reactions, the proposed methodology relies on the direct termination of living PEtOx chains by amino groups introduced onto the silica surface. Consequently, polymer immobilization is achieved without additional polymer functionalization, isolation of the polymer prior to grafting, or the use of dedicated coupling agents, thereby considerably simplifying the overall synthetic procedure while maintaining covalent attachment. To the best of our knowledge, this strategy has not previously been applied to the preparation of PEtOx-functionalized mesoporous silica particles. To investigate the influence of polymer chain length on the immobilization process, two well-defined PEtOx samples differing in molar mass were synthesized and subsequently grafted onto amino-functionalized mesoporous silica particles. Particular attention was paid to evaluating the effect of polymer chain length on the extent of polymer grafting and the resulting physicochemical properties of the hybrid materials.

**Scheme 1:**
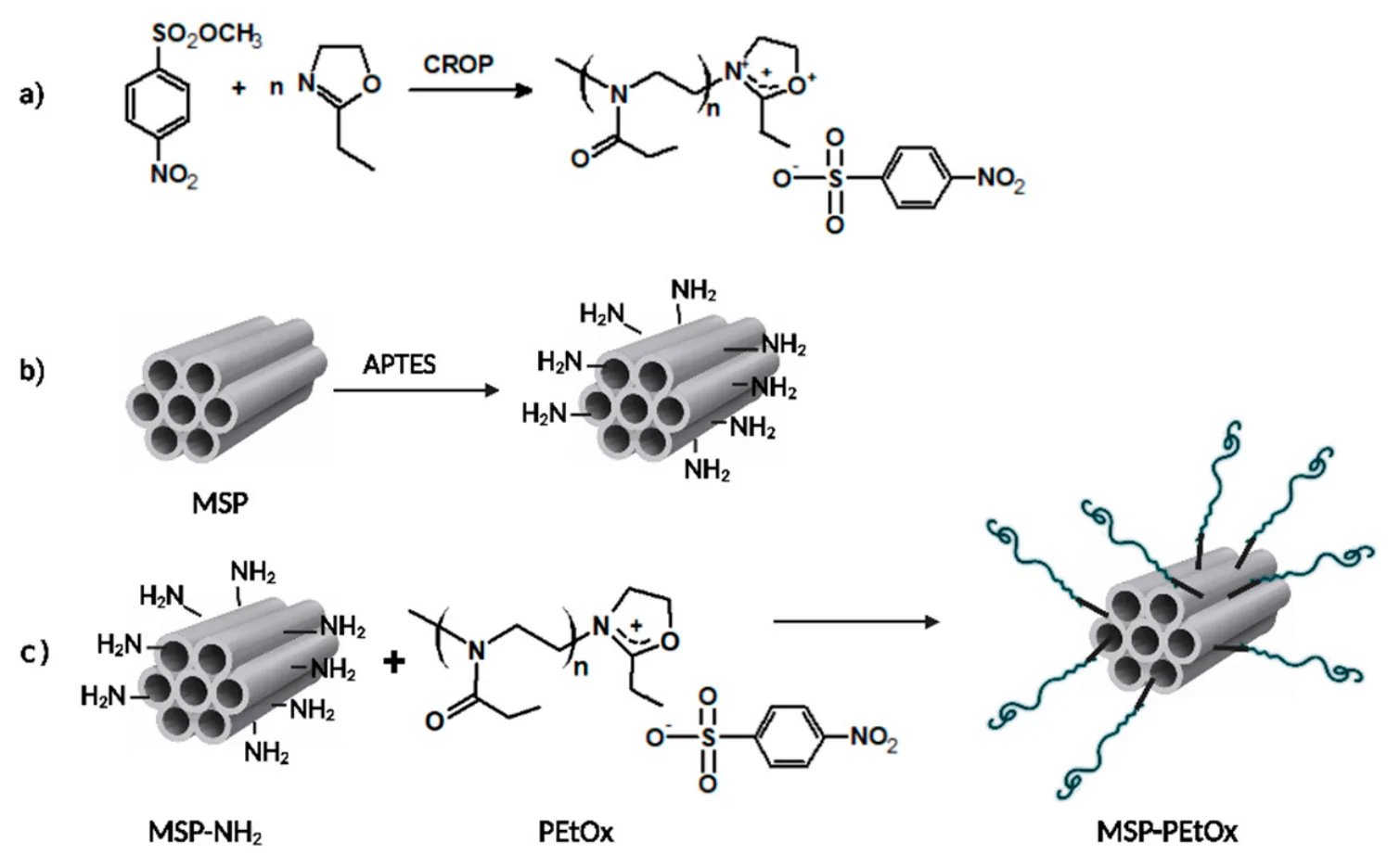
CROP of 2-ethyl-2-oxazoline (**a**), MSP modification using (3-aminopropyl)triethoxysilane (APTES) (**b**), and grafting of PEtOx to MSP-NH_2_ (**c**).

The synthetic pathway is illustrated in Scheme 1. First, well-defined PEtOx homopolymers were prepared by living CROP of 2-ethyl-2-oxazoline using methyl 4-nitrobenzenesulfonate as the initiator (Scheme 1a). In both experiments, the same molar amount of initiator and monomer concentration were used, and the polymerizations were carried out to complete monomer conversion. Since each initiator molecule generates one growing PEtOx chain in the living CROP process, the two polymerization mixtures contained the same theoretical number of living polymer chain ends prior to the grafting step. In parallel, mesoporous silica particles were functionalized with aminopropyl groups using APTES to introduce nucleophilic amino functionalities capable of terminating the living polymer chains (Scheme 1b). Finally, the same amount of amino-functionalized silica was added to each polymerization mixture after complete monomer conversion, enabling chain-end termination and covalent immobilization of PEtOx on the silica surface (Scheme 1c). Consequently, assuming comparable amino-group densities for the silica samples, the theoretical molar ratio of living PEtOx chain ends to surface amino groups was identical in the two experiments.

**Figure 1:**
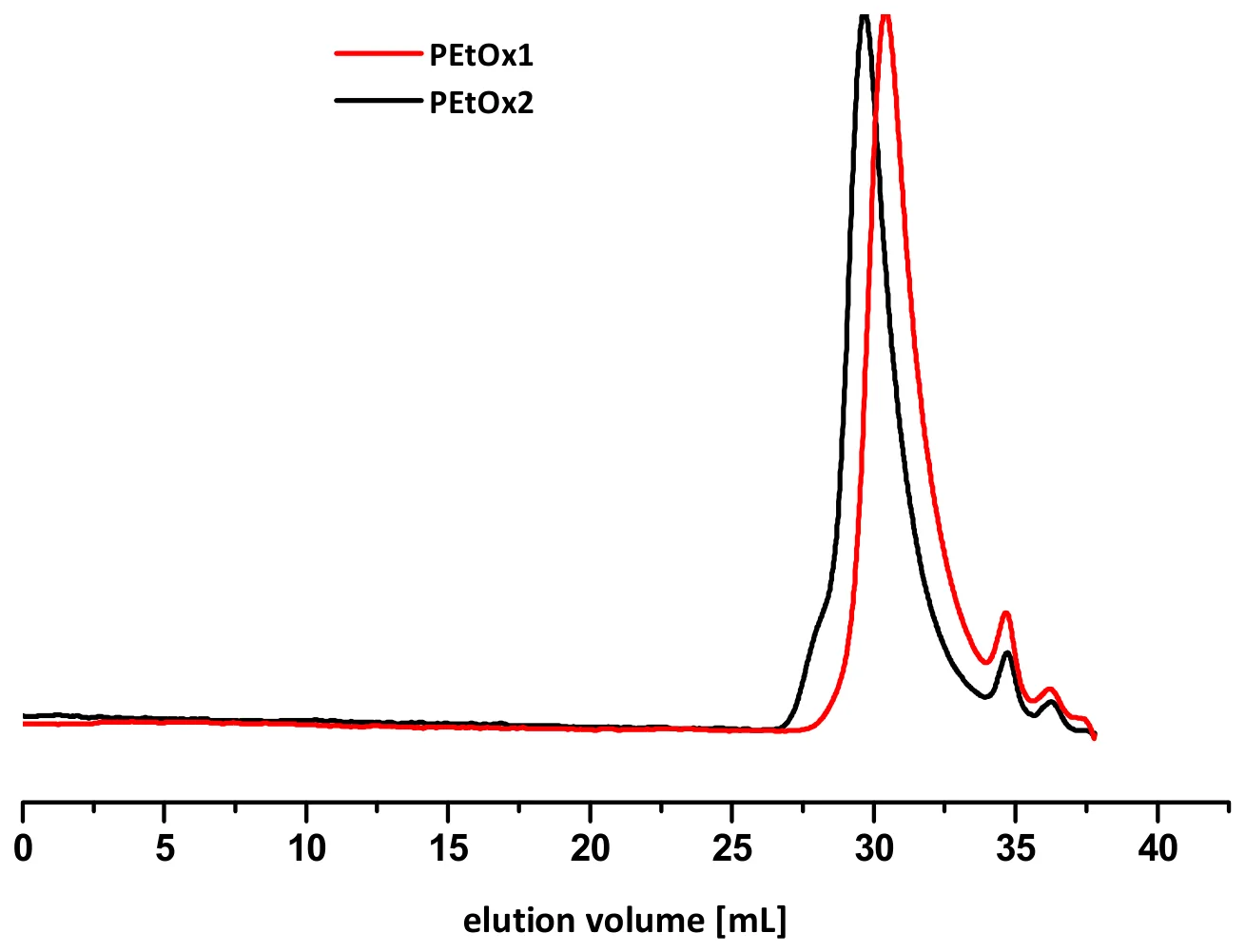
Chromatograms of obtained polymers (DMF, 1 mL/min).

GPC-MALLS analysis confirmed the successful synthesis of well-defined PEtOx homopolymers with number-average molar masses of approximately 5000 g mol^−1^ (PEtOx1) and 7500 g mol^−1^ (PEtOx2), both exhibiting narrow molar mass distributions (Đ = 1.12) (Figure 1). The experimental molar masses were in good agreement with the theoretical values, indicating control over the polymerization process.

The immobilization mechanism is based on the well-established susceptibility of living poly(2-oxazoline) chains to nucleophilic attack. Primary amino groups are known to efficiently terminate the cationic propagating species, forming stable covalent bond [41]. In the proposed strategy, amino groups anchored to the MSP surface simultaneously act as terminating agents and anchoring sites for the growing polymer chains. This concept eliminates the need for polymer isolation and introducing reactive end groups into the polymer prior to grafting, representing a significant simplification compared with conventional grafting-to methodologies. After complete reaction the cycles of extensive washing were applied and the hybrid particles were thoroughly characterized.

### 3.2. The Characterization of Polymer Grafted to MSP

**Figure 2:**
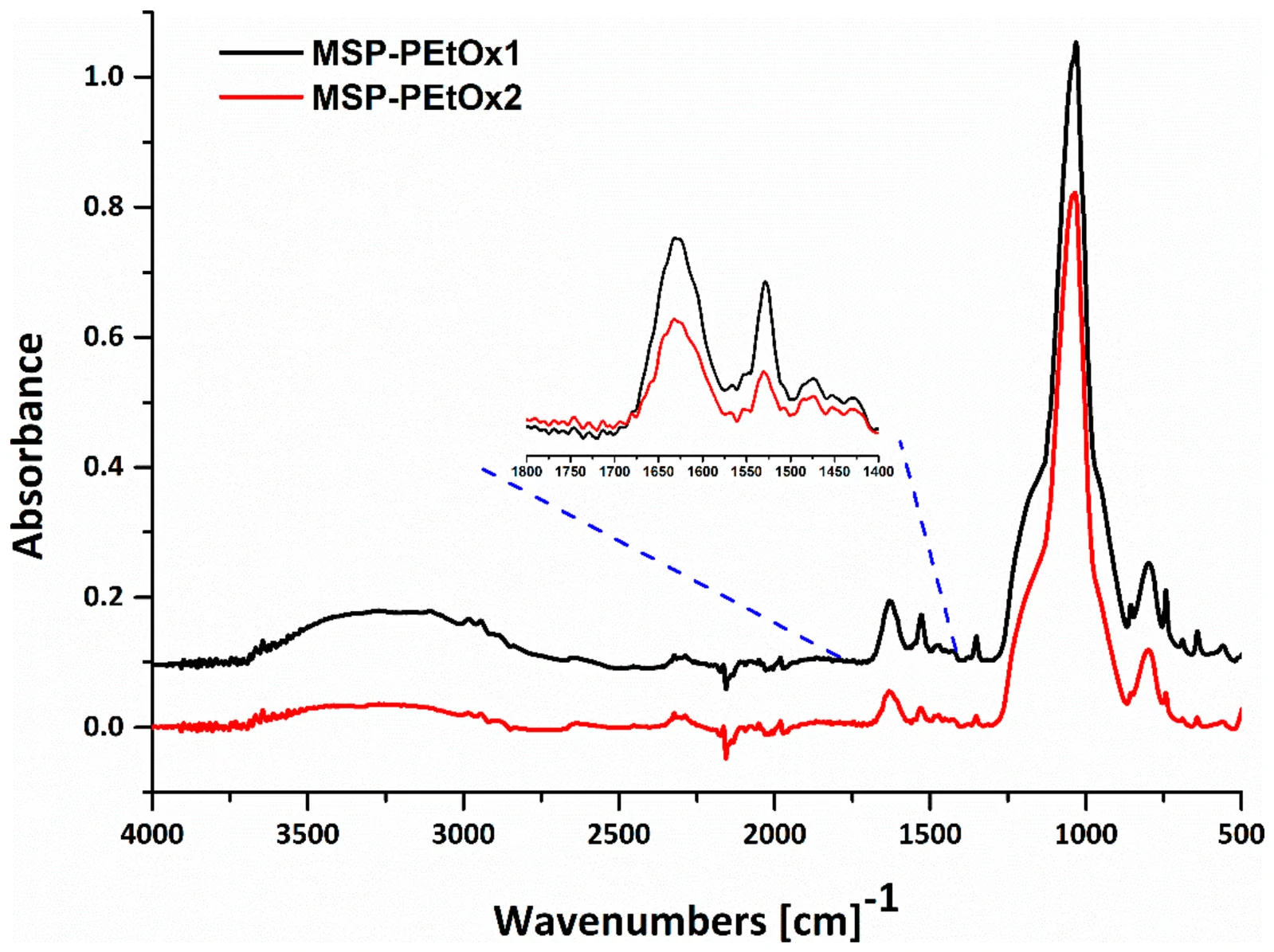
FT-IR spectra of MSP-PEtOx1 and MSP-PEtOx2.

Successful covalent immobilization of PEtOx onto MSP was first verified by ATR FT-IR spectroscopy. The spectra of initial MSP and amino-modified MSP-NH_2_ samples (Figure S1), confirm the presence of amino groups after modification. The asymmetric stretching vibrations of the Si–O–Si framework appeared at approximately 1090 cm^−1^ for both materials (not shown). Modification of the MSP sample with APTES resulted in the appearance of a band at 1507 cm^−1^, corresponding to the vibration of primary amine groups (–NH_2_) [42,43]. The band at 1550 cm^−1^ can be attributed to protonated amino groups (–NH_3_^+^), although the former may also be associated with the presence of adsorbed water. The formation of protonated amines may result from interactions with adsorbed water molecules or neighboring silanol groups. The bands observed between 1455 and 1360 cm^−1^ can be assigned to deformation vibrations of –CH_2_ and ethoxy groups. In addition, bands at 2970, 2932, and 2875 cm^−1^, characteristic of C–H stretching vibrations, were observed in the spectrum of the amino-modified sample. These findings are consistent with our previous studies, in which the successful incorporation of aminopropyl groups was also confirmed by solid-state NMR spectroscopy [44]. In the ATR FT-IR spectra of MSP-PEtOx1 and MSP-PEtOx2 (Figure 2), a characteristic absorption band is observed at approximately 1629 cm^−1^, which can be assigned to the C=O stretching vibration of the tertiary amide groups of PEtOx [7,34,45]. The absorption in the 1530–1550 cm^−1^ region may be associated with the surface amino functionalities introduced by APTES, including protonated amino species, and may be affected by the local surface environment after polymer immobilization [39,42,43]. In addition, following the grafting reaction, the formation of a secondary amine linkage between the active PEtOx chain end and the amino groups of APTES may also contribute to this region.

To exclude the presence of physically adsorbed PEtOx on the mesoporous silica, the control experiment with PEtOx terminated by n-propylamine (M_n_ = 5500 g mol^−1^) was performed. After washing, no detectable polymer presence on the silica surface was observed (Figure S2).

Additionally, the modification of the MSP-NH_2_ with polymer was confirmed by SEM-EDS, which analyses the surface of studied materials. Table 1 presents the elemental composition of MSP-NH_2_ and the representative polymer-coated sample MSP-PEtOx1.

MSP-NH_2_ shows a typical elemental composition for amine-bearing mesoporous silica [46]. Silicon and oxygen dominate; the composition is consistent with the silica framework (SiO_2_), while the presence of ~1.74 wt.% nitrogen confirms the successful incorporation of amino groups onto the silica surface through APTES coupling. After PEtOx grafting, a substantial increase in the carbon and nitrogen content is observed, accompanied by a notable decrease in Si contribution. Such changes confirm the successful introduction of polymer onto the silica surface.

### 3.3. Influence of Polymer Molar Mass on Grafting Efficiency

Having confirmed the successful covalent immobilization of PEtOx onto the mesoporous silica surface, the next objective was to determine whether the molar mass of the polymer affects the efficiency of the proposed immobilization strategy. Since the grafting mechanism relies on the reaction between the living polymer chain end and amino groups located on the silica surface, increasing polymer chain length may be expected to introduce steric constraints that limit the accessibility of reactive surface sites. To investigate this possibility, the influence of polymer chain length on grafting efficiency was first evaluated by elemental analysis (Table 2). Both hybrid materials exhibited considerably higher carbon and nitrogen contents than the parent MSP-NH_2_, confirming successful polymer immobilization. However, the increase was substantially greater for MSP-PEtOx1 than for MSP-PEtOx2. The carbon content increased from 4.41 wt.% for MSP-NH_2_ to 12.58 wt.% for MSP-PEtOx1, whereas a value of 8.64 wt.% was observed for MSP-PEtOx2. A similar trend was found for nitrogen, with contents increasing to 3.12 wt.% and 2.33 wt.% for MSP-PEtOx1 and MSP-PEtOx2, respectively. This suggests that PEtOx2 with higher molar mass (7500 g/mol) was successfully grafted to the MSP, albeit to a lesser extent than PEtOx1 (5000 g/mol). The difference between MSP-PEtOx1 and MSP-PEtOx2 probably is attributed to the length of the polymer chains and steric hindrance. These issues are important factors influencing the grafting process, although they should not be considered the key determinant of grafting efficiency.

**Figure 3:**
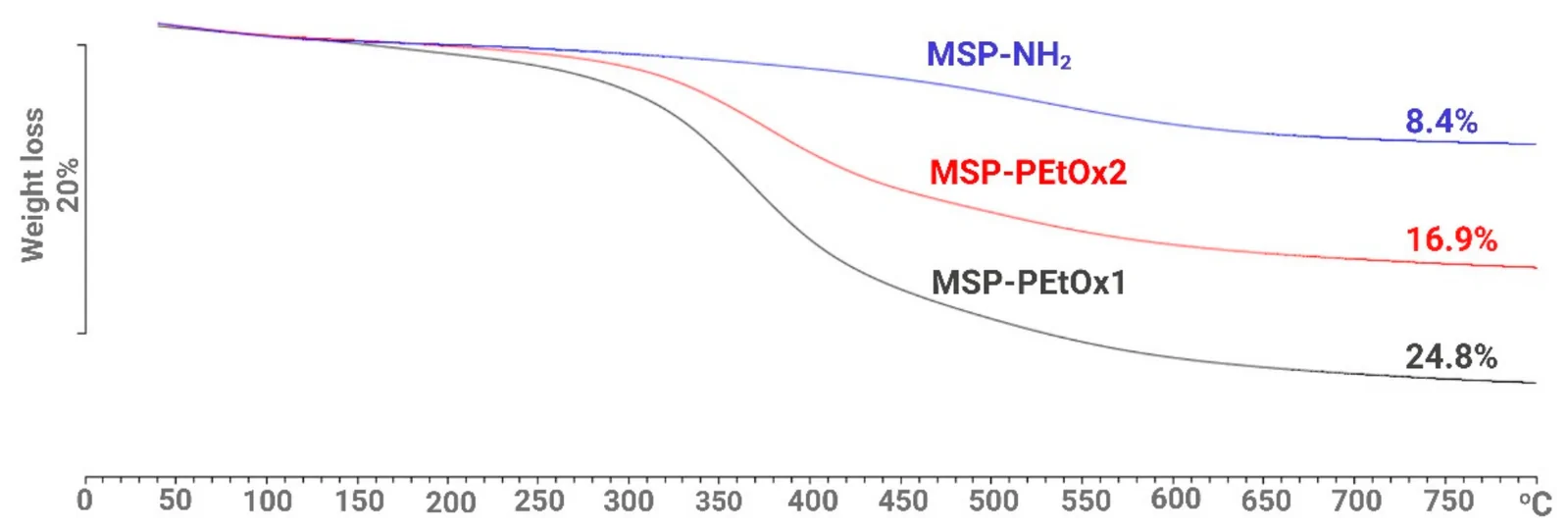
Thermogravimetric analysis of the MSP-NH_2_, MSP-PEtOx1 and MSP-PEtOx2.

Independent confirmation of these observations was provided by thermogravimetric analysis (Figure 3). While amino-functionalized silica exhibited an organic content corresponding to a weight loss of 8.4%, the hybrid materials displayed markedly higher values of 24.8% and 16.9% for MSP-PEtOx1 and MSP-PEtOx2, respectively. Taking into account the APTES presence on the silica particles, the weight loss of the hybrid materials was recalculated using the formula:

PEtOx content [wt.%] = (L_hybrid_ − L_MSP-NH2_)/(1 − L_MSP-NH2_) (1)

where

PEtOx content—mass fraction of PEtOx in the final hybrid material [wt.%];

L_hybrid_—weight loss of MSP-PEtOx1 or MSP-PEtOx2;

L_MSP-NH2_—weight loss MSP-NH_2_ = 0.084.

The calculated PEtOx contents were approximately 17.9 wt.% for MSP-PEtOx1 and 9.3 wt.% for MSP-PEtOx2, corresponding to polymer loadings of approximately 218 and 102 mg PEtOx per g of MSP-NH_2_, respectively.

Using above values, the experimentally determined number-average molar masses of PEtOx1 (5000 g mol^−1^) and PEtOx2 (7500 g mol^−1^) and the BET surface area of MSP-NH_2_ (365 m^2^ g^−1^), the apparent grafting densities were estimated to be approximately 0.072 and 0.022 chains nm^−2^ for MSP-PEtOx1 and MSP-PEtOx2, respectively [20]. These values represent apparent grafting densities normalized to the total BET surface area of MSP-NH_2_ and relatively inform that the shorter PEtOx1 chains are immobilized more efficiently than PEtOx2.

### 3.4. Effect of Polymer Grafting on Physicochemical Properties of MSP

Having established that PEtOx was successfully immobilized on the silica surface, the influence of the polymer layer on the physicochemical properties of the mesoporous support was subsequently investigated.

**Figure 4:**
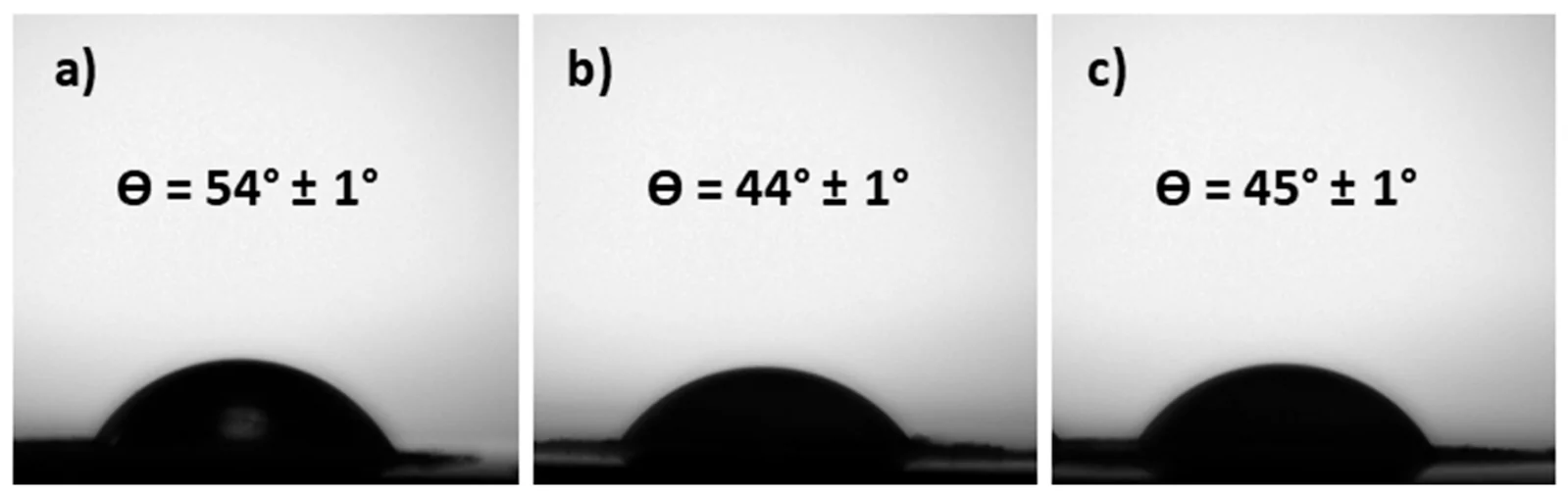
Water droplets on a silicon wafer with deposited MSP-NH_2_ (**a**), MSP-PEtOx1 (**b**), and MSP-PEtOx2 (**c**).

The BET specific surface area and pore characteristics of the tested samples estimated using nitrogen adsorption–desorption isotherms are shown in Table 2. The results showed that the amino-modified silica sample is characterized by a typically high specific surface area (365 m^2^ g^−1^) and a large pore volume (0.98 cm^3^ g^−1^), which is consistent with the literature values reported for mesoporous silica materials functionalized with aminopropyl groups [47]. After grafting of the PEtOx to the surface of MSP-NH_2_, a noticeable decrease in the specific surface area and total pore volume was observed. The BET specific surface area decreased to 263 m^2^ g^−1^ for MSP-PEtOx1 and to 306 m^2^ g^−1^ for MSP-PEtOx2, while the pore volume decreased to 0.66 cm^3^ g^−1^ and 0.81 cm^3^ g^−1^, respectively. Such reductions are well documented for mesoporous silica materials subjected to surface functionalization or polymer grafting [13]. They are typically attributed to (i) partial pore blocking by organic molecules anchored to the inner surface, (ii) occupation of mesopores by grafted polymer chains, and (iii) reduction in available surface area due to the formation of a conformal organic layer. Despite the reduction in surface area and pore volume, both MSP-PEtOx1 and MSP-PEtOx2 maintain an average pore diameter of about 10 nm, close to that of the parent MSP-NH_2_ (~10.71 nm). This indicates that the pores of the mesoporous structure were largely preserved after functionalization. Surface modification also altered the wettability of the particles. Contact angle measurements (Figure 4) showed a gradual decrease from 54° ± 1° for MSP-NH_2_ (Figure 4a) to 44–45° ± 1° for PEtOx-coated MSP (Figure 4b,c). The increased hydrophilicity originates from the presence of the highly hydrated PEtOx layer, which is capable of extensive hydrogen-bonding interactions with water molecules. Interestingly, despite the differences in apparent grafting density, both polymer-coated samples exhibited very similar contact angles, suggesting that even partial surface coverage is sufficient to dominate the interfacial properties of the particles.

**Figure 5:**
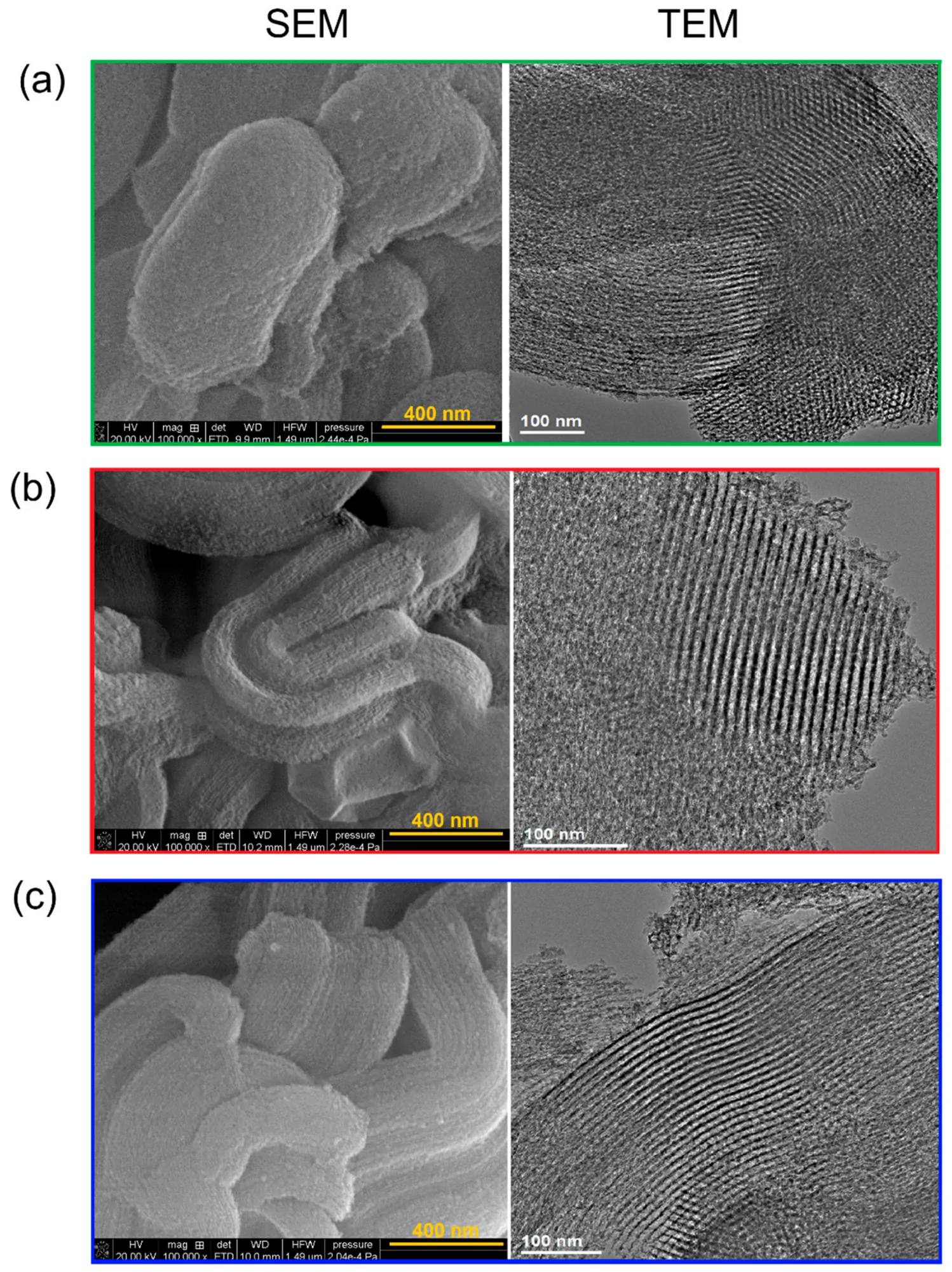
SEM and TEM micrographs of MSP-NH_2_ (**a**); MSP-PEtOx1 (**b**) and MSP-PEtOx2 (**c**).

To assess the changes in morphology of silica particles after modifications, scanning electron microscopy (SEM) and transmission electron microscopy (TEM) analysis was performed (Figure 5).

The micrographs of MSP-NH_2_ (Figure 5a) reveal typical rod or wheat-like morphology similar to unmodified SBA-15 [48]. TEM micrographs of MSP-NH_2_ show the characteristic, ordered hexagonal mesopore arrangement. The channels are clearly visible as parallel, uniform dark lines [48]. These observations demonstrate that the introduction of amine groups primarily alters surface chemistry without substantially affecting particle morphology, as was reported in the literature [47,48]. After polymer grafting (MSP-PEtOx1, Figure 5b, and MSP-PEtOx2, Figure 5c), the SEM micrographs showed a visibly increased surface roughness compared with the MSP-NH_2_. In the TEM images, some changes in the appearance of the mesoporous channels were also observed. No substantial differences in particle morphology were observed between MSP-PEtOx1 and MSP-PEtOx2.

## 4. Conclusions

A straightforward strategy for the preparation of poly(2-ethyl-2-oxazoline)-functionalized mesoporous silica particles was successfully developed by exploiting the direct termination of living cationic polymer chains with amino groups anchored on the silica surface. Comprehensive physicochemical characterization confirmed the successful immobilization of PEtOx while preserving the ordered mesoporous architecture with an average pore diameter of approximately 10 nm of the silica support. The complementary results obtained from FT-IR spectroscopy, elemental analysis, thermogravimetric analysis, nitrogen adsorption–desorption measurements, and electron microscopy consistently demonstrated the formation of a stable polymer layer on the particle surface. The use of two well-defined PEtOx samples of different molar masses revealed that polymer chain length plays a key role in the immobilization process. Shorter polymer chains achieved higher grafting efficiency than their higher molar mass counterparts, most likely due to reduced steric hindrance and improved accessibility of amino groups located on the silica surface. Consequently, polymer molar mass can be regarded as an effective parameter for controlling the degree of surface functionalization and the resulting physicochemical properties of the hybrid materials. The presented strategy not only expands the synthetic approaches available for the preparation of poly(2-oxazoline)-based hybrid materials but also opens the way for the immobilization of structurally and functionally diverse poly(2-oxazoline)s on three-dimensional porous particles. This approach creates new opportunities to exploit the broad spectrum of properties accessible within the poly(2-oxazoline) family, including stimuli-responsive, antifouling, antibacterial, and other biofunctional behavior, while simultaneously extending the application potential of mesoporous particles. Therefore, the resulting hybrid materials may find broad applications in areas such as biomedicine, separation, catalysis, and sensing.

## Figures and Tables

**Table 1:** SEM-EDS analysis of the measured samples.

Sample	Si [wt. %]	O [wt. %]	C [wt. %]	N [wt. %]
MSP-NH_2_	34.59 ± 2.69	50.58 ± 1.81	13.09 ± 1.38	1.74 ± 0.35
MSP-PEtOx1	24.64 ± 4.47	38.64 ± 5.51	33.20 ± 3.42	3.52 ± 0.36

**Table 2:** Characteristics of the studied systems.

Sample	N% ^1^	C% ^1^	H% ^1^	S_BET_^2^ [m^2^/g]	Langmuir Surface Area ^2^ [m^2^/g]	Vp ^2^ [cm^3^/g]	D ^2^ [nm]
MSP-NH_2_	1.51 ± 0.01	4.41 ± 0.04	1.509 ± 0.01	365 ± 24	544 ± 25	0.98 ± 0.02	10.71 ± 0.05
MSP-PEtOx1	3.12 ± 0.02	12.58 ± 0.11	2.416 ± 0.02	263 ± 15	391 ± 21	0.66 ± 0.01	10.04 ± 0.07
MSP-PEtOx2	2.33 ± 0.02	8.64 ± 0.03	2.224 ± 0.01	306 ± 17	455 ± 29	0.81 ± 0.01	10.54 ± 0.07

^1^ Determined from the elemental analysis; ^2^ surface properties of studied samples obtained from the nitrogen adsorption–desorption isotherms at −196 °C.

## Data Availability

The original contributions presented in this study are included in the article and the Supplementary Materials. Further inquiries can be directed to the corresponding author.

## Supplementary Materials

The following supporting information can be downloaded at: https://www.mdpi.com/article/10.3390/ma19173775/s1. Figure S1. FT-IR spectra of the initial MSP and the amino-modified MSP material and Figure S2. FT-IR spectrum of the MSP material after removal of the pre-terminated PEtOx chains.
