# Supplementary material for: Direct Immobilization of Living Poly(2-ethyl-2-oxazoline) Chains onto Mesoporous Silica: A Simplified Grafting-To Strategy for Hybrid Organic–Inorganic Materials
Source: Materials (Basel). 2026 Sep 4;19(17):3775. doi: 10.3390/ma19173775 (PMC13567434; doi:10.3390/ma19173775)
Supplement: Supplementary file 1 [file materials-19-03775-s001.zip › materials-4474952-supplementary.pdf]

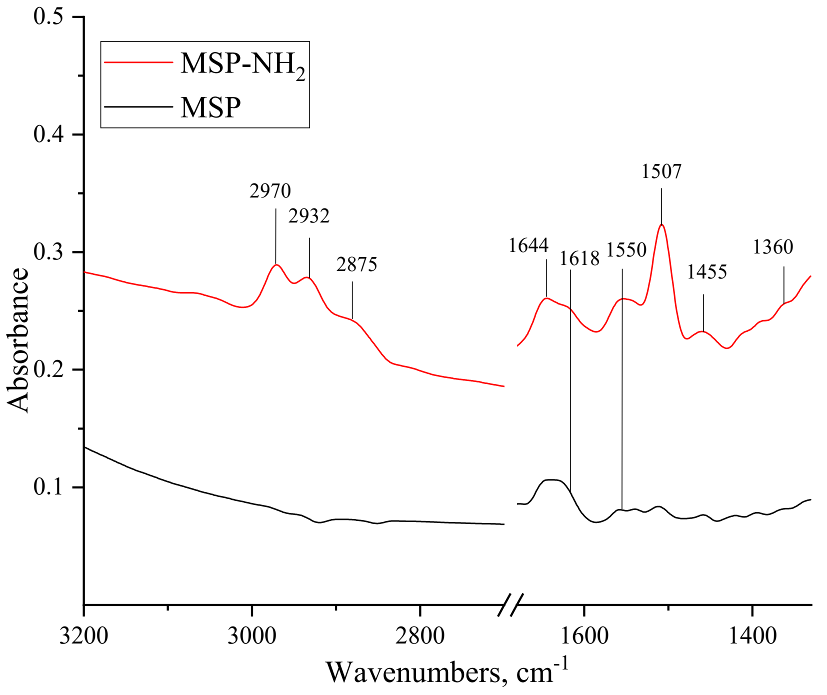

Figure S1. FT-IR spectra of the initial MSP and the amino-modified MSP material.

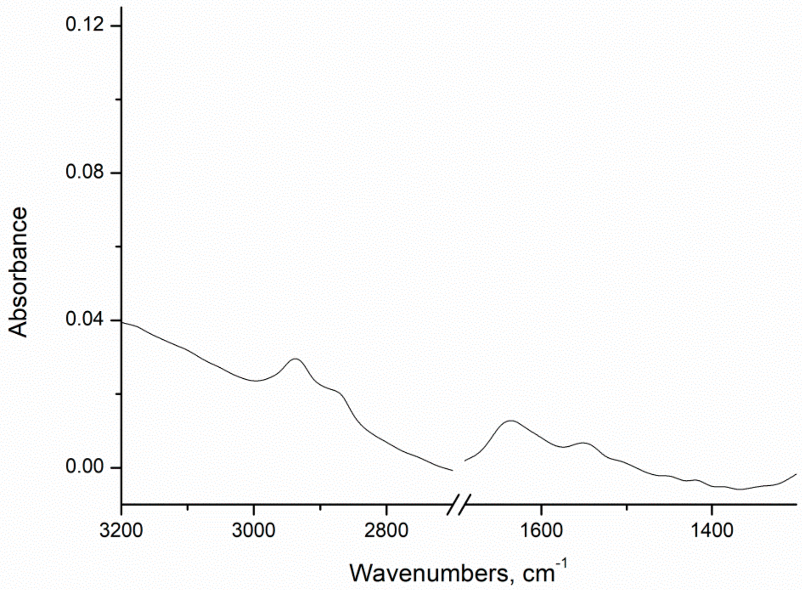

Figure S2. FT-IR spectrum of the MSP material after removal of the pre-terminated PEtOx chains.
